# The proteome of bacterial membrane vesicles in *Escherichia coli*—a time course comparison study in two different media

**DOI:** 10.3389/fmicb.2024.1361270

**Published:** 2024-03-06

**Authors:** Mia S. C. Yu, Dapi Menglin Chiang, Marlene Reithmair, Agnes Meidert, Florian Brandes, Gustav Schelling, Christina Ludwig, Chen Meng, Benedikt Kirchner, Christian Zenner, Laurent Muller, Michael W. Pfaffl

**Affiliations:** ^1^Division of Animal Physiology and Immunology, School of Life Sciences Weihenstephan, Technical University of Munich (TUM), Freising, Germany; ^2^Institute of Human Genetics, University Hospital, LMU Munich, Munich, Germany; ^3^Department of Biomedicine, University of Basel, Basel, Switzerland; ^4^Department of Anesthesiology, University Hospital, LMU Munich, Munich, Germany; ^5^Bavarian Center for Biomolecular Mass Spectrometry (BayBioMS), Technical University of Munich (TUM), Freising, Germany; ^6^Intestinal Microbiome, ZIEL – Institute for Food & Health, School of Life Sciences, Technical University of Munich (TUM), Freising, Germany; ^7^Department of Otorhinolaryngology, Head and Neck Surgery, University Hospital of Basel, Basel, Switzerland

**Keywords:** *Escherichia coli*, bacterial membrane vesicle, growth curve, bMVs, proteomics, functional assay, OMVs

## Abstract

**Introduction:**

Bacteria inhabit the in- and outside of the human body, such as skin, gut or the oral cavity where they play an innoxious, beneficial or even pathogenic role. It is well known that bacteria can secrete membrane vesicles (MVs) like eukaryotic cells with extracellular vesicles (EVs). Several studies indicate that bacterial membrane vesicles (bMVs) play a crucial role in microbiome-host interactions. However, the composition of such bMVs and their functionality under different culture conditions are still largely unknown.

**Methods:**

To gain a better insight into bMVs, we investigated the composition and functionality of *E. coli* (DSM 105380) bMVs from the culture media Lysogeny broth (LB) and RPMI 1640 throughout the different phases of growth (lag-, log- and stationary-phase). bMVs from three time points (8 h, 54 h, and 168 h) and two media (LB and RPMI 1640) were isolated by ultracentrifugation and analyzed using nanoparticle tracking analysis (NTA), cryogenic electron microscopy (Cryo-EM), conventional transmission electron microscopy (TEM) and mass spectrometry-based proteomics (LC–MS/MS). Furthermore, we examined pro-inflammatory cytokines IL-1β and IL-8 in the human monocyte cell line THP-1 upon bMV treatment.

**Results:**

Particle numbers increased with inoculation periods. The bMV morphologies in Cryo-EM/TEM were similar at each time point and condition. Using proteomics, we identified 140 proteins, such as the common bMV markers OmpA and GroEL, present in bMVs isolated from both media and at all time points. Additionally, we were able to detect growth-condition-specific proteins. Treatment of THP-1 cells with bMVs of all six groups lead to significantly high IL-1β and IL-8 expressions.

**Conclusion:**

Our study showed that the choice of medium and the duration of culturing significantly influence both *E. coli* bMV numbers and protein composition. Our TEM/Cryo-EM results demonstrated the presence of intact *E. coli* bMVs. Common *E. coli* proteins, including OmpA, GroEL, and ribosome proteins, can consistently be identified across all six tested growth conditions. Furthermore, our functional assays imply that bMVs isolated from the six groups retain their function and result in comparable cytokine induction.

## 1 Introduction

Extracellular vesicles (EVs) are vesicles with a lipid bilayer and a size ranging from 30 to 1,000 nm that are produced by all domains of life (Brown et al., 2015; Abels and Breakefield, 2016). Depending on their origin, their content can consist of various proteins (both intracellular and transmembrane proteins), nucleic acids and lipids (Abels and Breakefield, 2016; Ebner and Götz, 2019). Moreover, they play an important role in intercellular communication not only between different species but even amongst different kingdoms (Brown et al., 2015; Abels and Breakefield, 2016; van Niel et al., 2018; Diaz-Garrido et al., 2021).

Bacterial extracellular vesicles, also called bacterial membrane vesicles (bMVs) (Zhu et al., 2021; Hosseini-Giv et al., 2022), were firstly discovered in gram-negative bacteria in the 1960s and in gram-positive bacteria in the 1990s (Chatterjee and Das, 1967; Brown et al., 2015). In the past years, the interest in investigating bMVs has increased as they can be utilized in medicine for both preventive and diagnostic applications such as vaccination or drug delivery platforms or as potential biomarkers for, e.g., cancer detection (Ünal et al., 2011; Chronopoulos and Kalluri, 2020; Zhu et al., 2021; Hosseini-Giv et al., 2022). Moreover, the bMVs of gram-negative bacteria (also called outer membrane vesicles or OMVs) with their associated proteins were shown to not only serve as delivery vehicles and nucleators for biofilm formation but also as factors for bacterial survival and virulence (Kulp and Kuehn, 2010). EVs isolated from human samples or cell culture media by different isolation methods lead to different results in vesicle size distribution, particle number and protein amount (Tauro et al., 2012; Van Deun et al., 2014; Lobb et al., 2015; Helwa et al., 2017; Veerman et al., 2021). Furthermore, vesicle contamination due to media ingredients is widely observed in human cell culture (Pham et al., 2021). The extent of vesicle contamination for the isolation of bMVs from bacterial culture media remains poorly understood. Lysogeny broth (LB) is commonly used in other studies for cultivation of *Escherichia coli* (*E. coli*) (Bielaszewska et al., 2017; Blackburn et al., 2021; Reimer et al., 2021) while RPMI 1640 represents an originally eukaryotic growth medium that has been proposed to better reflect the physiological situation in a patient than classical bacterial growth media (Hill et al., 2019; Meerwein et al., 2020). Moreover, according to our analysis, RPMI 1640 does not contain any detectable particles as opposed to LB. As bMVs play a role in cell-cell-communication, their composition might be impacted by different growth conditions such as different culture media. However, little is known about this aspect.

The aim of this study was to compare if and how the composition as well as the functionality of bMVs obtained from pathogenic *E. coli* (strain: DSM 105380) changed over time when being cultured in different media. Furthermore, a comprehensive proteomic characterization can help to identify quantitative, qualitative, morphological and protein biomarkers. These proteins can serve as candidates in future studies for bMV based biomarkers in bacteremia, sepsis or other *E. coli* induced diseases.

## 2 Methods

### 2.1 Bacterial strain, cultivation, and determination of the growth curve

All following investigations were performed using the *E. coli* strain DSM 105380 obtained from the German Collection of Microorganisms and Cell Cultures (DSMZ). Cultures were maintained on tryptone soy agar (TSA) (Carl Roth GmbH + Co. KG, Karlsruhe, Germany; CP70.1) up to passage 4 at 37°C. For the experiment, liquid cultures were prepared by transfer of one colony to 30 mL of LB (Becton, Dickinson and Company, Franklin Lakes, United States; BD 244620) and 30 mL of RPMI 1640 (PAN-Biotech GmbH, Aidenbach, Germany; P04-16518), respectively, and incubated at 37°C for 2 days.

The growth curve was determined by measurement of the optical density at 600 nm (OD_600_) in 250 mL of either LB or RPMI 1640 in three replicates. Two bottles were prepared for each medium and incubated at 37°C with one being inoculated with 10 mL of liquid culture and one being untreated to serve as a control. Measurements of the OD_600_ were performed with 1 mL of culture against blank medium that was stored at 4°C at the following time points after starting the incubation: 0 h, 2 h, 8 h, 24 h, 30 h, 48 h, 54 h, 72 h, 96 h, and 168 h. Visualization of the growth curve was performed using Python 3 (Van Rossum and Drake, 2009).

### 2.2 Sample collection and bacterial membrane vesicle isolation

A bacterial growth curve generally consists of four different phases: (Abels and Breakefield, 2016) lag phase, (Brown et al., 2015) logarithmic phase, (Ebner and Götz, 2019) stationary phase, and (Diaz-Garrido et al., 2021) death phase followed by a long-term stationary phase (Pletnev et al., 2015). The exact sampling times as indicated above were evaluated in detail by various growth experiments in advance (data not shown). With the lag phase being characterized by very low bacterial growth due to the time the bacterial cells need in order to adapt to the conditions of their new environment (Pin and Baranyi, 2008; Pletnev et al., 2015), we chose 2 h as the first time point for bacterial culture medium collection to represent the late lag phase and the beginning of the logarithmic phase. 8 h bacterial culture medium was collected as the middle of the logarithmic phase and 54 h as the stationary phase.

It was previously shown that, under laboratory conditions, *E. coli* cultures can remain viable for years even after the nutrients contained in the medium are exhausted, entering a state known as the long-term stationary phase, without showing a significant death phase (Kram and Finkel, 2014; Takano et al., 2023). Thus, we chose 168 h representing the late stationary phase as the last collection point. 250 mL of *E. coli* culture medium were incubated in a nonpyrogenic 500 mL Erlenmeyer flask (Corning Incorporated, Corning NY, United States; Costar^®^ 430,422) at 37°C and collected at 2 h, 8 h, 54 h, and 168 h while shaking at 120 rpm. *Escherichia coli* culture media were collected and subsequently filtered using a 0.22 μm PES filter (Merck KGaA, Darmstadt, Germany; S2GPT02RE). All sample supernatants were stored at −20°C in 250 mL nonpyrogenic bottles (Corning Incorporated; Costar^®^ 8,390) until further processing. Three biological replicates were collected on different days.

The bMVs were isolated from 30 mL of the collected 250 mL of filtered supernatant using ultracentrifugation at 4°C and 150,000 rcf. The isolation volume was split in a total of three ultracentrifugation steps of 2 h, each, followed by a washing step with 10 mL of phosphate buffered saline (PBS) (Sigma + Merck, Darmstadt, Germany; D8537) for 2 h using 14 mL endotoxin-free ultracentrifugation tubes (Beckman Coulter GmbH, Krefeld, Germany; C14294). Pellets were subsequently resuspended in 35 μL of PBS for further NTA and functional assays and in 35 μL of 1X Radioimmunoprecipitation assay (RIPA) buffer (Abcam plc, Cambridge, United Kingdom; ab156034) for the proteomics testing.

### 2.3 Nanoparticle tracking analysis

Particle numbers and sizes were determined by ZetaView^®^ Nanoparticle Tracking Analyzer PMX 110 (Particle Metrix GmbH, Inning am Ammersee, Germany) both before and after bMV isolation. The supernatant and the isolated bMVs were diluted in PBS to a final volume of 1 mL if needed. For each measurement, two measurement cycles were performed by scanning 11 positions each and acquiring 60 frames per second under the following settings: pre-acquisition parameters were set to: (Abels and Breakefield, 2016) a sensitivity of 80, (Brown et al., 2015) shutter to 70, (Ebner and Götz, 2019) cell temperature to 23°C, and (Diaz-Garrido et al., 2021) trace length to 15. After recording, the videos were analyzed with the built-in ZetaView^®^ software 8.05.11 SP1 (Particle Metrix GmbH) with the following analysis parameters: minimum particle brightness: 20; minimum size of 5 pixels; maximum size of 1,000 pixels and particle size distribution (PSD) nm/class of 10 and PSD classes/decade of 10.

### 2.4 Transmission electron microscopy

A total of 5 μL of the undiluted sample were adsorbed for 60 s to glow-discharged parlodion/carbon-coated copper grids (Electron Microscopy Sciences, Hatfield, United States; FCF200-NI). The grids were then blotted, washed three times with ddH_2_O and negatively stained on two droplets of 2% uranyl acetate solution (Merck KGaA, Darmstadt, Germany). Samples were imaged using a FEI Talos F200C TEM (FEI/Thermo Fisher Scientific Inc., Waltham, United States) operated at 120 kV. Electron micrographs were recorded on a Veleta Camera (EMSIS GmbH, Muenster, Germany).

### 2.5 Cryogenic electron microscopy

The cryo-EM sample preparation followed our previous publication (Benecke et al., 2022). A total of 4 μL aliquot of each sample was adsorbed onto holey carbon-coated grid (Ted Pella Inc., Redding, United States) blotted with Whatman 1 filter paper and vitrified into liquid ethane at −178°C using a Leica GP plunger (Leica Microsystems GmbH, Wetzlar, Germany). Frozen grids were transferred onto a Talos electron microscope (FEI/Thermo Fisher Scientific Inc., Waltham, United States) using a Gatan 626 cryo-holder (Gatan, Inc., Pleasanton, United States). Electron micrographs were recorded at an accelerating voltage of 200 kV, using a low-dose system (20 e−/Å^2^) and keeping the sample at low temperature. Micrographs were recorded on a CETA camera (Thermo Fisher Scientific Inc., Waltham, United States).

### 2.6 Protein preparation for proteomics

20 μL of bMVs were mixed with 20 μL 2X RIPA buffer (Abcam plc, Cambridge, United Kingdom; ab156034) and stored at −80°C. Next, samples were boiled at 70°C for 10 min and sonicated at 4°C for 5 min. The sonicated protein extracts were centrifuged at 10,000 rcf at 4°C for 30 min. Protein concentration was measured by performing a Bicinchoninic acid assay (Thermo Fisher Scientific Inc., Waltham, United States). 2 μg of proteins were further mixed with ddH_2_O and 6 μL of 4× Laemmli Sample Buffer (Bio-Rad Laboratories, Hercules, United States) with 2-mercaptoethanol (Merck KGaA, Darmstadt, Germany) in a final volume of 24 μL. 24 μL of bMVs proteins with 1× Laemmli Sample Buffer were sent to the proteomics core facility (Bavarian Center for Biomolecular Mass Spectrometry).

### 2.7 Mass spectrometry-based proteomics

In accordance with standard procedures, in-gel trypsin digestion of all bMV samples was performed (Shevchenko et al., 2006). Briefly, the samples were separated on a Nu-PAGE^™^ 4–12% Bis-Tris protein gel (Thermo Fisher Scientific Inc., Waltham, United States) for about 1 cm. Subsequently, the accumulated and not size-separated single protein band per sample was cut out, reduced (50 mM dithiothreitol), alkylated (55 mM chloroacetamide), and digested overnight with trypsin (Trypsin Gold, mass spectrometry grade, Promega). The dry peptide samples were resuspended in 25 μL buffer A (2% acetonitrile, 0.1% formic acid in HPLC grade water), of which 5 μL were injected per mass spectrometry (MS) measurement.

LC–MS/MS measurements were carried out on a Dionex Ultimate 3,000 RSLCnano system coupled to a Q-Exactive HF-X mass spectrometer (Thermo Fisher Scientific Inc., Waltham, United States). Injected peptides were delivered to a trap column (ReproSil-our C18-AQ, 5 μm, Dr. Maisch, 20 mm × 75 μm, self-packed) at a flow rate of 5 μL/min in 100% solvent A (0.1% formic acid in HPLC grade water). After 10 min of loading, peptides were transferred to an analytical column (ReproSil Gold C18-AQ, 3 μm, Dr. Maisch, 450 mm × 75 μm, self-packed) and separated using a 50 min gradient from 4 to 32% of solvent B (0.1% FA, 5% DMSO in acetonitrile) in solvent A (0.1% FA, 5% DMSO in HPLC grade water) at 300 nL/min flow rate. The Q-Exactive HF-X mass spectrometer was operated in data-dependent acquisition and positive ionization mode. MS1 spectra (360–1,300 m/z) were recorded at a resolution of 60 k using an automatic gain control target value of 3,000,000 and a maximum injection time of 45 ms. Up to 18 peptide precursors were selected for fragmentation. Only precursors with charge states 2 to 6 were selected, and dynamic exclusion of 25 s was enabled. Peptide fragmentation was performed using higher-energy collision-induced dissociation and normalized collision energy of 26%. The precursor isolation window width was set to 1.3 m/z. MS2 Resolution was 15,000 with automatic gain control target value of 100,000 and a maximum injection time of 25 ms (complete proteome).

### 2.8 Label-free quantification of proteins

Peptide identification and quantification were performed using the software MaxQuant (version 1.6.3.4) (Tyanova et al., 2016) with its built-in search engine Andromeda (Cox et al., 2011). MS2 spectra were searched against the *E. coli* (reference strain K12) proteome from Uniprot (UP000000625, downloaded at 2022.12.08) supplemented with common contaminants (built-in option in MaxQuant). Trypsin/P was specified as a proteolytic enzyme, and carbamidomethylated cysteine was set as a fixed modification. Oxidation of methionine and acetylation at the protein N-terminus were defined as variable modifications. Results were adjusted to a 1% false discovery rate on peptide spectrum match level and protein level employing a target-decoy approach using reversed protein sequences. LFQ intensities were further analyzed using Perseus version v2.0.10.0 (Tyanova et al., 2016). We used the built-in filter functions of Perseus to filter out reverse, only identified by site and contaminant proteins and log10 transformed all protein intensities (Tyanova et al., 2016).

To filter for proteins reproducibly detectable over all media and time we used the function “Filter rows based on valid values” and set this to “3 in each group” (one group being defined as biological triplicates from one growth condition). Venn diagram analysis stacked bar graphs were created using Prism version 10.1.1. We found 140 proteins to be detected in all 6 tested growth conditions. The log10 LFQ intensities of these 140 proteins were further analyzed with NormFinder (version 0.953) (Andersen et al., 2004).

For determination of differential proteins between groups, we filtered the data using the function “Filter rows based on valid values” and set this to “3 in at least one group.” Missing values were imputed by “normal distribution” (default Perseus setting: width = 0.3, down shift = 1.8). In order to obtain Principal component analysis (PCA) and volcano plot, we performed a multiple sample test: ANOVA (adjusted with Benjamin-Hochberg, FDR: 0.05). PCA was generated with the ANOVA tested dataset. Volcano plots were further plotted with additional multiple-hypothesis adjusted *t*-test (two-sided, number of randomizations: 250, FDR: 0.05 and S0: 0.1). The generation of volcano plots involved plotting the log10 fold change on the *x*-axis and the −log10 *p*-values on the *y*-axis for each pairwise comparison. The most significantly different proteins per pairwise comparison were selected based on fold change, lowest *q*-value in ANOVA and lowest *p*-value in *t*-test. Enrichment analysis of significant proteins against the gene ontology cellular component (GOCC) terms in the ANOVA testing dataset was conducted using Fisher’s Exact test (Benjamini-Hochberg FDR truncation with a threshold value of 0.0001).

### 2.9 Functional assay

1 × 10^5^ cells of the monocytic cell line THP-1 were seeded in 200 μL of a 96-well plate at a density of 5 × 10^5^ cells/mL. 1 × 10^7^ particles of bMVs were diluted first in 5 μL of cell culture medium (RPMI 1640 medium containing 10% fetal bovine serum and 1% penicillin/streptomycin). The diluted bMVs were used to treat the THP-1 cells in a 100:1 ratio at 37°C and 7% CO_2_ for 24 h. 100 ng/mL of Lipopolysaccarides (LPS) from *E. coli* O127:B8 (Sigma + Merck; L4516-1MG) served as a positive control. Concentrations of Interleukin-1β (IL-1β) (Catalog #: DY201, R&D Systems, Minneapolis, MN, United States) and IL-8 (Catalog #: DY208, R&D Systems, Minneapolis, MN, United States) in culture supernatants were measured by ELISA according to the manufacturer’s instructions (R&D Systems, Minneapolis, MN, United States).

### 2.10 Statistical analysis

Prism version 10.1.1 (GraphPad Software, Boston, United States) was used to statistically analyze (Abels and Breakefield, 2016) the growth curve results with a mixed-effects model (REML) as well as a Tukey’s multiple comparisons test, (Brown et al., 2015) the NTA results with a mixed-effects model (REML) as well as a Tukey’s multiple comparisons test, (Ebner and Götz, 2019) the number of detected proteins in proteomics was analyzed using one-way ANOVA with Tukey’s multiple comparisons test and (Diaz-Garrido et al., 2021) the ELISA assay with an ordinary one-way ANOVA with Dunnett’s multiple comparisons test. Furthermore, the proteomics results were statistically analyzed by ANOVA and student’s *t*-test in Perseus version 2.0.10.0 (Max-Planck Institute of Biochemistry) (Tyanova et al., 2016). The specific test used is described in the respective figure legend.

## 3 Results

### 3.1 Characterization of bacterial growth rate and particle events in culture

#### 3.1.1 Bacterial growth rate

**Figure 1:**
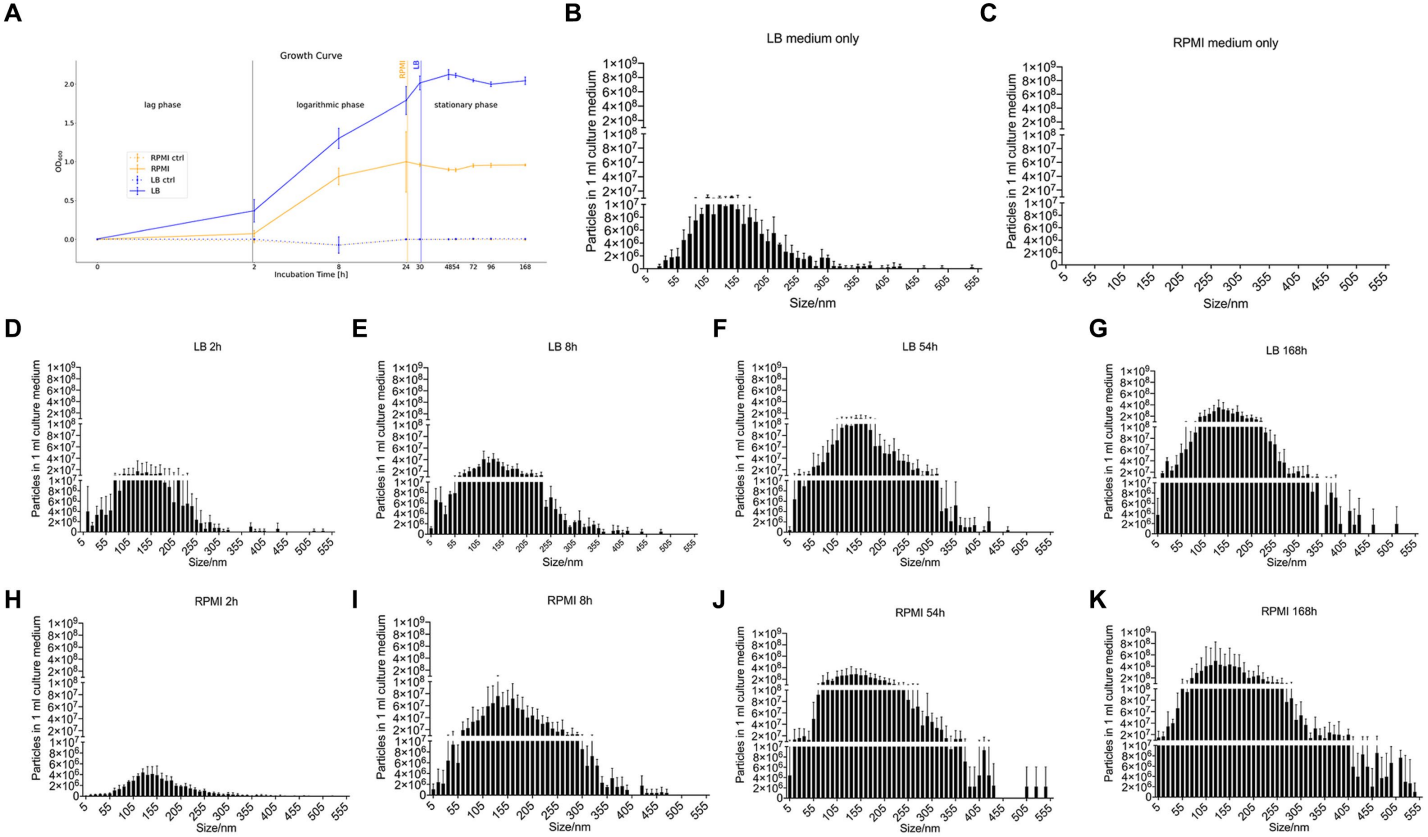
Growth curve of *E. coli* and nanoparticle size distribution of *E. coli* culture supernatant. Growth curve of *E. coli* (DSM 105380) that was obtained by measuring the optical density at 600 nm wavelength in RPMI 1640 and LB as well as the respective control media without added bacteria (RPMI ctrl and LB ctrl) **(A)**. Bar plot represents mean ± SD of three biological replicates of the particle numbers and size distribution of nanoparticles in culture medium only or *E. coli* culture supernatant, LB medium only **(B)**, RPMI 1640 medium only **(C)**, LB_2h **(D)**, LB_8h **(E)**, LB_54h **(F)**, LB_168h **(G)**, RPMI_2h **(H)**, RPMI_8h **(I)**, RPMI_54h **(J)**, and RPMI_168h **(K)**. Particles and size distribution were measured with ZetaView^®^. Significance was calculated by mixed-effects model (REML) and Tukey’s multiple comparisons test by Prism version 10.1.1.

The control media which were incubated without bacteria inoculation were at a constant OD_600_ 0 ± 0.005 (Figure 1A). For the media containing bacteria, it is recognizable that the OD_600_ in LB rose faster and earlier than the OD_600_ in RPMI 1640 (Figure 1A). In LB, the plateau phase was reached after 24 h while in RPMI 1640, this phase was already obtained after 8 h (Figure 1A). The OD_600_ measurements at 8 h, 24 h, 30 h, 48 h, 54 h, 72 h, 96 h, and 168 h in both LB and RPMI cultures were significantly higher compared to their respective measurements at 0 h and 2 h (Supplementary Tables S1, S2).

#### 3.1.2 Particle events in bacterial culture medium

The number of particles and their sizes were evaluated by NTA at the different incubation time points for the two tested media before bMV isolation.

Pure LB medium contains medium-borne particles while no particles could be detected in pure RPMI 1640 (Figures 1B,C). In general, the number of particles detected in 1 mL of medium increased with longer incubation periods both in LB (Figures 1D–G) and RPMI 1640 (Figures 1H–K). Besides, we detected bigger particle sizes and higher yields in RPMI 1640 compared to LB at all time points except for 2 h. Especially at the later time points, incubation in LB generated less small (<10 nm) and less big (>355 nm) particles. The distribution of particle sizes and yield can be seen in Figures 1B–K.

Moreover, when performing a mixed-effects model as well as a Tukey’s test with the distribution of particle size to particle number, the differences between each treatment were significant (value of *p* <0.05) except for LB_168h vs. RPMI_54h (not significant, value of *p* = 0,9,742) (Supplementary Table S3).

### 3.2 Quantity, morphology, and common protein surface markers of bacterial membrane vesicles

#### 3.2.1 Particle events of bacterial membrane vesicles

As the NTA measurement 2 h after incubation showed the lowest particle numbers in 1 mL medium for both media, we excluded this time point for further experiments. This decision is based on the natural decrease of particles after bMV isolation which would most likely have reached the technical particle detection limit. Thus, the following experiments were performed for time points 8 h, 54 h, and 168 h.

**Figure 2:**
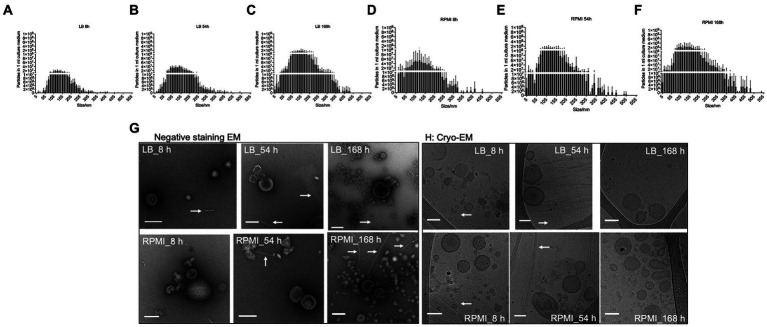
Size distribution, morphology and common surface markers of *E. coli* bMVs. Bar plot represents mean ± SD of three biological replicates of the particle numbers and size distribution of *E. coli* bMVs of LB_8h **(A)**, LB_54h **(B)**, LB_168h **(C)**, RPMI_8h **(D)**, RPMI_54h **(E)**, and RPMI_168h **(F)**. The replicates were collected on three different days and further processed together on the same day. Particle numbers and size distribution were measured with ZetaView^®^. Significance was calculated by mixed-effects model (REML) and Tukey’s multiple comparisons test by Prism version 10.1.1 and can be withdrawn from Supplementary Table S1. Morphology of all six groups of *E. coli* bMVs was determined with TEM **(G)** and Cryo-EM **(H)**. Arrows indicate the presence of flagella in the *E. coli* bMV preparations.

The number of particles and their sizes were evaluated by NTA at the different incubation time points for the two tested media after bMV isolation. In general, the number of particles detected in 1 mL of isolated bMVs increased with longer incubation periods both in LB (Figures 2A–C) and RPMI 1640 (Figures 2D–F). Interestingly, we found higher yields in RPMI 1640 compared to LB at all time points. The median sizes of bMVs were similar in all six groups (LB_8h: 135.2 nm ± 3.7 nm, LB_54h: 139.0 nm ± 4.9 nm, LB_168h: 143.6 nm ± 10.4 nm, RPMI_8h: 131.1 nm ± 9.4 nm, RPMI_54h: 130.7 nm ± 4.7 nm, and RPMI_168h: 134.8 nm ± 1.6 nm). The distribution of particle sizes and yield can be seen in Figures 2A–F. Moreover, when performing a mixed-effects model as well as a Tukey’s test with the distribution of particle size to particle number the differences between each treatment were significant (value of *p* <0.05) except for LB_168h vs. RPMI_54h (not significant, value of *p* = 0.8606) (Supplementary Table S4).

#### 3.2.2 Morphology of bacterial membrane vesicles

Purified *E. coli* bMVs were observed as intact cup-shaped membrane vesicles with a size range of 50–200 nm in TEM (Figure 2G). These results were concordant with our Cryo-EM findings, which showed intact lipid bilayer membrane structures with a size range of 50–150 nm (Figure 2H). Interestingly, flagellum-like structures can be found at all time points and in both media in Cryo-EM as well as TEM (Figure 2H).

### 3.3 Comparison of *Escherichia coli* membrane vesicle protein profiles

#### 3.3.1 Proteins consistently found in bMVs across media and time points

The chaperonin protein (GroEL) and the outer membrane protein A (OmpA) are widely recognized markers in various bMVs (Kim et al., 2008; McCaig et al., 2013; Daleke-Schermerhorn et al., 2014; Reimer et al., 2021). Additionally, the protein composition across the two specific culture conditions remained unknown. Therefore, we investigated the proteome composition of *E. coli* bMVs isolated from two different media and the three time points (LB_8h, LB_54h, LB168h, RPMI_8h, RPMI_54h, and RPMI_168h) as described above using LC–MS/MS based proteomics.

**Figure 3:**
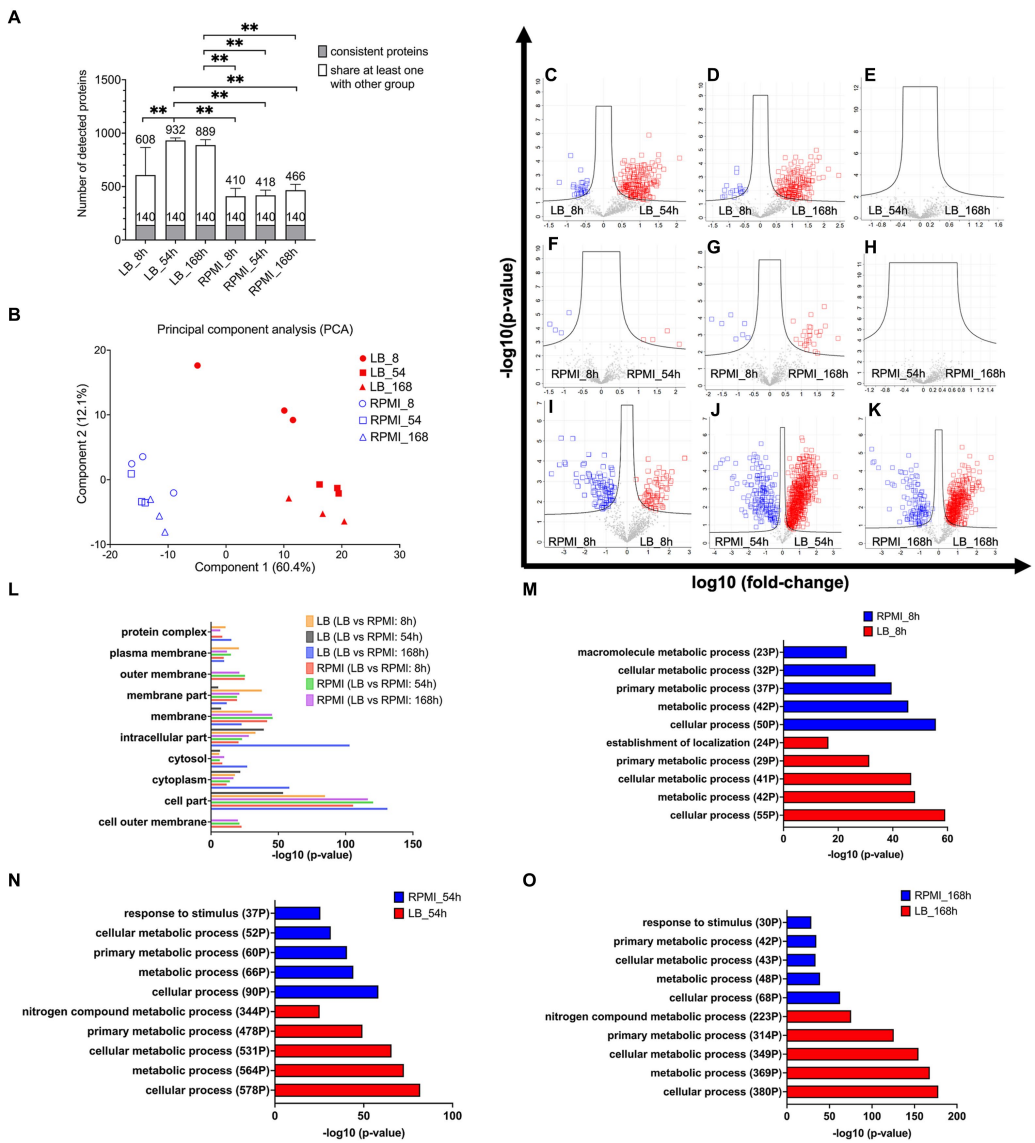
Protein composition in *E. coli* bMVs and their associated pathway. Stacked bar plot shows mean ± SD of number of detected proteins, the gray bar indicates the 140 consistent proteins, and the white bar indicates proteins shared with at least one other group with filter criteria for three biological replicates **(A)**. Principal Component Analysis (PCA) to compare the protein composition of six different groups **(B)**. Volcano plot shows log-transformed fold-change of each protein plotted against the log-transformed fold-change. Significantly different proteins between LB_8h and LB_54h **(C)**, LB_8h and LB_168h **(D)**, LB_54h and LB_168h **(E)**, RPMI_8h and RPMI_54h **(F)**, RPMI_8h and RPMI_168h **(G)**, RPMI_54h and RPMI_168h **(H)**, RPMI_8h and LB_8h **(I)**, RPMI_54h and LB_54h **(J)**, and RPMI_168h and LB_168h **(K)**. Significance was calculated by ANOVA and student’s *t*-test in Perseus version 2.0.10.0 (Max-Planck Institute of Biochemistry). The proteins exhibiting significantly higher levels and over two-fold changes were presented in either the blue or red squares on the volcano plots. Bar diagram of enrichment analysis (two-sided Fisher’s Exact Test, FDR = 0.0001) of gene ontology cellular component (GOCC) pathways of high abundant bMV-associated proteins in the comparison of the same time points between LB and RPMI 1640 **(L)**, gene ontology biological process (GOBP) pathways of high abundant bMV-associated proteins in the comparison of 8 h **(M)**, 54 h **(N)**, and 168 h **(O)** between LB and RPMI 1640 which were involved in *E. coli* biological process pathways. Significance levels for the different enrichment classes are displayed as −log10 *p*-values.

The data was filtered for proteins that were consistently observed in biological triplicates across all six groups (comprising three time points and two media). We identified the following numbers of bMV-associated proteins with their average detection values ± standard deviation: 608 ± 257 in LB_8h culture, 932 ± 24 in LB_54h, 889 ± 51 in LB_168h, 410 ± 74 in RPMI_8h, 418 ± 48 in RPMI_54h, and 466 ± 54 in RPMI_168h. The stacked bar plot (Figure 3A) illustrates the identification of 140 bMV-associated proteins found consistently across all six groups. We also observed a significant increase in the number of bMV-associated proteins in LB_54h and LB168h compared to the three RPMI 1640 time points (RPMI_8h, RPMI_54h, and RPMI_168h) (Figure 3A, statistical calculation in Supplementary Table S5). Adapting the NormFinder algorithm (Andersen et al., 2004), we identified the top 10 consistently stable expressed proteins over all six groups, with 30S ribosomal protein S5 (rpsE) exhibiting the lowest stability value of 0.015 (see ranked 140 among proteins in Supplementary Table S6). Notably, the ribosomal proteins rpsE, rpsJ, rplS, rplF, rpsG, rplM, rpsM, and rplV consistently appeared among the top 9 stable expressed proteins across all six groups. Heat shock protein 60 (groL/GroEL) displayed a low stability value of 0.025, and OmpA had a stability value of 0.054 (see ranked 140 among proteins in Supplementary Table S6).

Principal component analysis (PCA) confirmed differences in the composition of *E. coli* bMV-associated proteins between LB and RPMI 1640 culture conditions (Figure 3B). Our proteomic results substantiated the consistent identification of GroEL and OmpA across all experimental conditions and are accordant with previous literature (Kim et al., 2008; McCaig et al., 2013; Daleke-Schermerhorn et al., 2014; Reimer et al., 2021). Importantly, while distinct protein compositions were observed in different culture media, this variation was not evident across the three time points.

#### 3.3.2 *Escherichia coli* bMV-associated proteins with significant abundance changed over time in LB medium

To unravel proteomic distinctions across different media and time points, we applied ANOVA and student *t*-tests to pinpoint proteins exhibiting significant differences in each combination (Figures 3C–K). In LB_8h, *E. coli* bMV-associated proteins demonstrated a substantial over 2-fold increase in 30 proteins (Figure 3C, see Supplementary Table S7) when compared to LB_54h. LB_54h, on the other hand, exhibited a significant over 2-fold increase in 204 proteins (Figure 3C, see Supplementary Table S7) when compared to LB_8h. Notably, OmpA displayed a significant increase in LB_8h when compared to LB_54h. In LB_8h, 18 proteins (Figure 3D, see Supplementary Table S7) were significantly elevated by over 2-fold when compared to LB_168h. Conversely, LB_168h exhibited 185 proteins (Figure 3D, see Supplementary Table S7) significantly increased by over 2-fold when compared to LB_8h. When comparing LB_54h and LB_168h, no significantly different proteins were identified in either group (Figure 3E).

#### 3.3.3 *Escherichia coli* bMV-associated proteins with significant abundance changed over time in RPMI 1640

The PCA results indicated similarities in the protein compositions of *E. coli* bMV-associated proteins across RPMI_8h, RPMI_54h, and RPMI_168h culture conditions (Figure 3B). As anticipated, only four *E. coli* bMV-associated proteins (malonyl CoA-acyl carrier protein transacylase FabD, isochorismate synthase EntC, membrane-bound lytic murein transglycosylase B MltB and putative uncharacterized protein YdfE) exhibited a significant increase of 2-fold changes in RPMI_8h (Figure 3F, protein lists see Supplementary Table S7) when compared to RPMI_54h. Further four proteins (RNA-binding protein Hfq, 50S ribosomal protein L20 RplT, negative modulator of initiation of replication SeqA and uncharacterized protein YqjD) showed elevated levels in RPMI_54h (Figure 3F, protein lists see Supplementary Table S7). In RPMI_8h, seven proteins (50S ribosomal protein L27 rpmA, 30S ribosomal protein S19 rpsS, branched-chain-amino-acid aminotransferase IlvE, isochorismate synthase EntC, 30S ribosomal protein S17 RpsQ, thiol:disulfide interchange protein DsbG and putative uncharacterized protein YdfE) (Figure 3G, protein lists see Supplementary Table S7) exhibited a significant increase of over 2-fold changes when compared to RPMI_168h. Conversely, RPMI_168h represented 26 proteins (Figure 3G, protein lists see Supplementary Table S7) that displayed a significant increase of over 2-fold changes when compared to RPMI_8h. No significantly different proteins were found within any group when comparing RPMI_54h and RPMI_168h (Figure 3H).

#### 3.3.4 Significant changes in abundance of *Escherichia coli* bMV-associated proteins across different media at the same time point

RPMI_8h samples revealed a significant increase of more than 2-fold changes in 96 proteins (Figure 3I, protein lists see Supplementary Table S7) associated with *E. coli* bMVs, when compared to LB_8h. Conversely, LB_8h samples displayed a significant increase of over 2-fold changes in 71 proteins (Figure 3I, protein lists see Supplementary Table S7) when compared to RPMI_8h. In RPMI_54h, a total of 180 proteins (Figure 3J, protein lists see Supplementary Table S7) exhibited a significant increase of over 2-fold changes when compared to LB_54h. On the other hand, LB_54h samples demonstrated a significant increase of over 2-fold changes in 694 proteins (Figure 3J, protein lists see Supplementary Table S7) when compared to RPMI_54h. For RPMI_168h, 119 proteins (Figure 3K, protein lists see Supplementary Table S7) showed a significant increase of over 2-fold changes when compared to LB_168h. In contrast, LB_168h exhibited 450 proteins (Figure 3K, protein lists see Supplementary Table S7) with a significant increase of over 2-fold changes when compared to RPMI_168h.

#### 3.3.5 Comparison of gene ontology analysis between the two media at the same time points

Remarkably, OmpA demonstrated a significant increase in RPMI_54h and RPMI_168h when compared to LB (Figures 3J,K). GOCC enrichment analysis (FDR = 0.0001) revealed that the outer membrane pathway (parent term in GOCC: membrane) is exclusively enriched in RPMI 1640 groups but not in LB groups (see Figure 3L). Furthermore, the pathways of cytoplasm, cytosol (parent term in GOCC: cytoplasm), intracellular part, and membrane were identified in all six groups (Figure 3L). Outer membrane pathway (parent term in GOCC: membrane) was exclusively enriched in LB_8h when compared to LB_54h and LB_168h (Supplementary Figure S1A and GO lists in Supplementary Table S8). In contrast, cytoplasm and cytosol (parent term in GOCC: cytoplasm) pathways were enriched in LB_54h and LB_168h when compared to LB_8h (Supplementary Figure S1A and GO lists in Supplementary Table S8). Membrane and cytoplasm pathways were exclusively enriched in RPMI_168h when compared to RPMI_8h (Supplementary Figure S1B and GO lists in Supplementary Table S8). The outer membrane (parent term in GOCC: membrane), plasma membrane (parent term in GOCC: membrane), and cytosol (parent term in GOCC: cytoplasm) pathways showed no differences in two comparisons: RPMI_54h compared to RPMI_8h, and RPMI_168h compared to RPMI_8h (Supplementary Figure S1B and GO lists in Supplementary Table S8).

Cellular process, metabolic process, cellular metabolic process, and primary metabolic process of gene ontology biological process (GOBP) pathway were all identified in pairwise comparisons between RPMI 1640 and LB groups (Figures 3M–O). However, LB groups exhibited more pronounced regulation of these four pathways. Specifically, for the most important pathway (cellular process), LB_8h had 55 identified proteins compared to 50 in the RPMI_8h. In LB_54h, 578 proteins were identified, contrasting with a mere 90 in the RPMI_54h. Similarly, in LB_168h, 380 proteins were identified compared to only 68 in the RPMI_168h (Figures 3M–O). Notably, the “response to stimulus” pathway was identified among the top 5 GOBP pathways in RPMI_54h (37 proteins) and RPMI_168h (30 proteins) but was conspicuously absent in the top 5 pathways of LB groups (Figures 3N–O). In contrast, the “nitrogen compound metabolic process” featured in the top 5 GOBP pathways of LB_54h (344 proteins) and LB_168h (223 proteins) but was absent in the top 5 pathways of RPMI groups (Figures 3N–O). Surprisingly, three proteins associated with the negative regulation pathway (negative regulation of cellular metabolic process, cellular process, macromolecule biosynthetic process, metabolic process, and macromolecule metabolic process) were identified in RPMI_54h, contrasting with RPMI_8h (Supplementary Figure S1C and GO lists in Supplementary Table S8). Figure 3 indicates that bMV-associated proteins are detectable in both membrane and cytosolic pathways across all six groups. Additionally, in the RPMI 1640 groups, bMV-associated proteins show a heightened representation in outer membrane pathways (RPMI_8h, RPMI_54h, and RPMI_168h). However, gene ontology analysis identified only a few proteins in the RPMI 1640 groups. Our analysis of GOBP pathways indicated that consistent basic biological functions such as cellular processes, metabolic processes, cellular metabolic processes, and primary metabolic processes were present in the bMVs across both the two different media and the three distinct time points.

### 3.4 Pro-inflammatory cytokines secretion in human monocytic THP-1 cells by treatment with *Escherichia coli* bMVs

**Figure 4:**
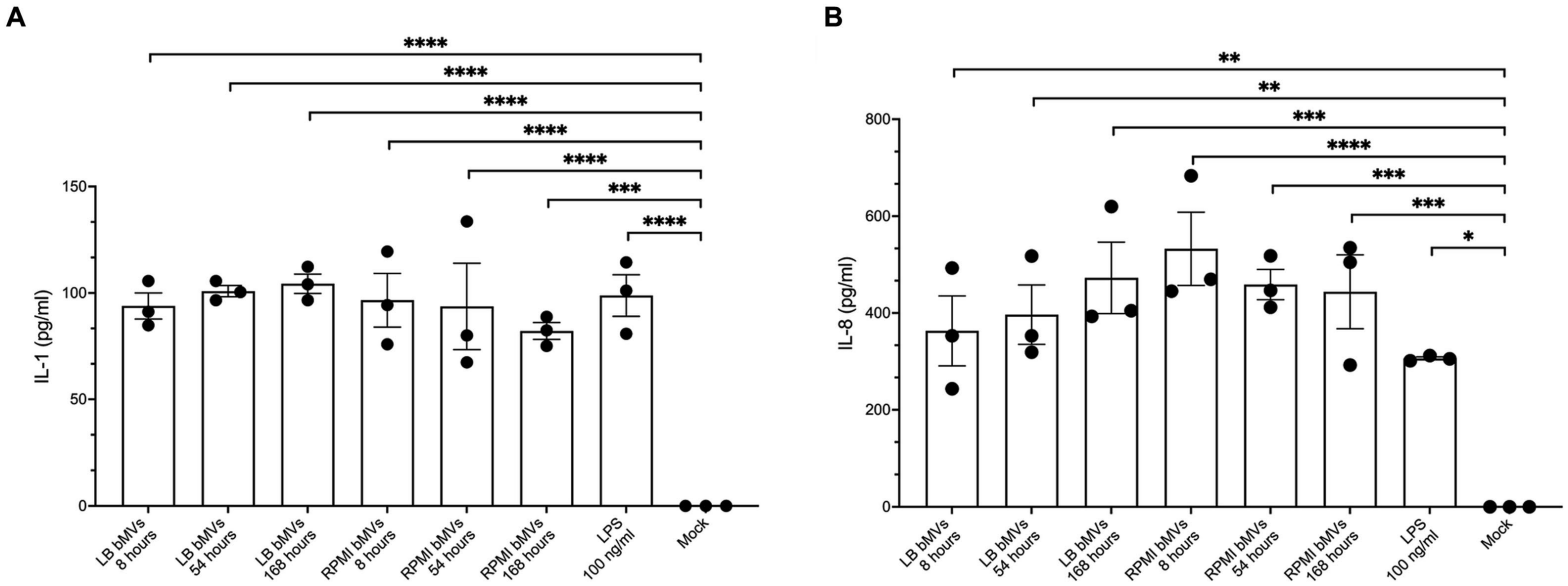
Pro-inflammatory cytokine secretion in human monocytic THP-1 cells by treatment with *E. coli* bMVs. A total of 1 × 10^7^ *E. coli* bMV particles was treated with 1 × 10^5^ human monocytic THP-1 cells (ratio 100:1) for 24 h at 37°C. Interleaved scatter with bars plot shows number of cytokines/chemokines IL-1β **(A)** and IL-8 **(B)** in pg./mL in the supernatant. ELISA assay was statistically analyzed by ordinary one-way ANOVA with Dunnett’s multiple comparisons test by Prism version 10.1.1 (GraphPad Software, Inc.). Data are presented as mean values ± SEM. (*) if *p* < 0.05, (**) if *p* < 0.0021, (***) if *p* < 0.0002, and (****) if *p* < 0.0001 was considered to indicate a statistically significant difference according to the GP style of Prism version 10.1.1 (GraphPad Software, Inc.).

bMVs from various bacterial species have been shown to induce pro-inflammatory cytokines such as IL-1β and IL-8 in monocytes/macrophages (Cecil et al., 2017; Lertjuthaporn et al., 2022). To understand whether *E. coli* bMVs can induce inflammatory cytokine secretion, we treated the human monocytic cell line THP-1 with bMVs of the different time points at a ratio of 100:1 (5 × 10^5^ cells with 5 × 10^7^ bMVs). Our results showed that IL-1ß was significantly higher in treatments with LB_8h bMVs (93.88 ± 10.65 pg./mL, *p* < 0.0001), LB_54h bMVs (100.86 ± 4.55 pg./mL, *p* < 0.0001), LB_168h bMVs (104.32 ± 7.85 pg./mL, *p* < 0.0001), RPMI_8h bMVs (96.55 ± 21.89 pg./mL, *p* < 0.0001), RPMI_54h bMVs (93.66 ± 35.12 pg./mL, *p* < 0.0001), and RPMI_168h bMVs (82.10 ± 6.88 pg./mL, *p* = 0.0001) compared to negative controls (Figure 4). 100 ng/mL of LPS served as a positive control and showed a significant induction of IL-1β secretion in THP-1 (98.80 ± 16.93 pg./mL, *p* < 0.0001). However, we did not observe any difference between the different treatments.

IL-8 was also significantly higher in treatments with LB_8h bMVs (362.98 ± 125.12 pg./mL, *p* = 0.025), LB_54h bMVs (396.52 ± 106.28 pg./mL, *p* = 0.0011), LB_168h bMVs (472.51 ± 127.87 pg./mL, *p* = 0.0002), RPMI_8h bMVs (532.67 ± 131.01 pg./mL, *p* < 0.0001), RPMI_54h bMVs (458.89 ± 54.11 pg./mL, *p* = 0.0003), and RPMI_168h bMVs (443.96 ± 132.17 pg./mL, *p* = 0.0004) compared to negative controls (Figure 4). 100 ng/mL of LPS served as a positive control and showed a significant induction of IL-8 secretion in THP-1 (306.19 ± 5.50 pg./mL, *p* = 0.01). Interestingly, the treatment with bMVs seemed comparable to the treatment with LPS only. However, we did not observe any major difference between different treatments.

## 4 Discussion

### 4.1 Enhanced *Escherichia coli* bMV secretion and protein cargo transition from logarithmic to stationary phase within LB or RPMI 1640 medium

In our study, particle numbers increased over time in both investigated *E. coli* culture media. This observation is in concordance with Zavan et al. who reported a significant increase in the production of *Helicobacter pylori* (*H. pylori*) bMVs over time in brain heart infusion broth (Zavan et al., 2019). Furthermore, they observed a narrower distribution of size among *H. pylori* bMVs, indicating reduced heterogeneity in size as the time progressed from 16 h to 48 h and eventually to 72 h (Zavan et al., 2019). However, Melo et al. reported that there were no significant differences in the size distribution of *H. pylori* bMVs between the 48 h, 64 h, and 72 h cultures (Melo et al., 2021). They did observe an increase in the particle number at 64 h compared to 48 h, but no notable difference was found between 64 h and 72 h (Melo et al., 2021). While we were unable to determine size differences in *E. coli* bMVs across the two different media and three time points, we found a similar increase in particle number.

Furthermore, their study demonstrated a significant increase in IL-8 cytokine secretion in human gastric adenocarcinoma cells when treated with late time points (48 h and 72 h) of *H. pylori* bMVs, whereas no such increase was observed at 16 h (Zavan et al., 2019). However, we did not observe comparable size alterations in *E. coli* bMVs, nor did we observe any functional changes in THP-1 cells upon treatment with different time points/media of *E. coli* bMVs. The difference in our treatment approach was that we fixed the number of particles instead of fixing the protein amounts (Zavan et al., 2019) in the functional assay. It is important to note that the same protein amounts do not necessarily equate to the same particle numbers, as they can be influenced by contaminating proteins or EV biogenesis (Webber and Clayton, 2013; Koliha et al., 2016). Future studies will be essential to clarify the relative relevance of fixing protein amounts versus particle numbers in a physiological setting. Yamanashi et al. reported that there were no differences in size and protein concentration observed in *Staphylococcus aureus* (*S. aureus*) bMVs from culture conditions at 6 h, 17 h, and 24 h (Yamanashi et al., 2022). Our *E. coli* bMVs across the six groups demonstrated similarities in terms of size and protein compositions. However, there is a limited number of studies that have specifically explored the time-dependent aspects in different bacterial strains. Future studies should consider investigating these factors in other bacterial strains as well.

### 4.2 Consistent protein cargo loading in LB and RPMI 1640 culture conditions

GroEL and OmpA have been reported as potential surface markers in *E. coli* bMVs (Kim et al., 2008; Daleke-Schermerhorn et al., 2014; Jiang et al., 2022). Our proteomics results also showed that GroEL and OmpA were consistent proteins in all samples with GroEL even being amongst the top 10 consistent proteins (Figure 3). This suggests that our results are comparable to previous findings and that these two proteins can be used as suitable biomarkers in *E. coli* bMVs. In addition, Ye et al. demonstrated that treatment with the antibiotic Imipenem increased bMV secretion in *Klebsiella pneumoniae* (*K. pneumoniae*), accompanied by elevated GroEL expression (Ye et al., 2021). Furthermore, treatment of macrophage cell lines with bMVs from wild type or Imipenem-treated *K. pneumoniae* induced pyroptotic responses and cytokine secretions, including IL-1β, IL-18, and tumor necrosis factor alpha (TNF-α) (Ye et al., 2021). Zhu et al. have demonstrated that the lectin-like oxidized low-density lipoprotein receptor-1 (LOX-1) on macrophages functions as the receptor for GroEL, which is essential for *E. coli* adhesion (Zhu et al., 2013). Our results showed that GroEL exhibited stable expression in all groups and had similar effects on the pro-inflammatory cytokines IL-1β and IL-8 in monocytic THP-1 cells. Further studies are required to explore whether GroEL is the necessary element that enables *E. coli* bMVs to stimulate IL-1β and IL-8 and/or enables the entry into monocytes/macrophages through LOX-1.

Ribosomal proteins represent over 70% of the top 20 out of the 140 consistent proteins in our study. Moreover, a consistent expression of the following GOBP pathways was identified across all conditions: cellular process, metabolic process, cellular metabolic process, and primary metabolic process (FDR: 0.0001). Chiu et al. also observed that 30S ribosomal subunit proteins were expressed in the proteomics of *E. coli* bMVs (Chiu et al., 2022). Kesavan et al. investigated the bMV proteins of another gram-negative bacterial species, *Acinetobacter baumannii*, revealing that bMVs might support bacterial survival through ribosomal proteins, chaperones, stress proteins and proteases (Kesavan et al., 2020). The increased bMV quantities during later growth phases, coupled with the identification of ribosomal proteins in our proteomic analysis, suggest that ribosomal proteins originating from *E. coli* bMVs might contribute to bacterial survival or communication.

### 4.3 Different protein cargo loading and protein pathways in LB and RPMI 1640 culture conditions

Previous studies showed that the cargo of bMVs exhibits significant variation across different growth conditions, leading to diverse biological outcomes (Zavan et al., 2019; Bitto et al., 2021; Yamanashi et al., 2022; Suzuki et al., 2023). Our findings further confirm this phenomenon, as we identified only 140 consistently expressed bMV-associated proteins across all investigated time points and medium combinations, while detecting a maximum of 870 distinct bMV-associated proteins in the sample with the greatest protein diversity (Figure 3). Interestingly, the GOCC enrichment analysis (FDR = 0.0001) revealed that the outer membrane pathway (part of membrane in GOCC) was exclusively identified in the RPMI 1640 groups compared to the LB groups (Figure 3N). Moreover, we observed a higher abundance of bMVs in the RPMI 1640 culture condition compared to LB. OmpA was also higher in RPMI_54h and RPMI_168h compared to LB. Taken together, our results suggest that bMV biogenesis and secretion may be enhanced in the RPMI 1640 culture condition. Previous studies showed that absence of Lpp, Tol-Pal complex proteins (Pal, TolA, and TolB) in gram-negative bacteria significantly increased the bMV secretion (Deatherage et al., 2009; Schwechheimer et al., 2013; Schwechheimer and Kuehn, 2015). In our study, TolB showed significantly higher levels in RPMI_8h, RPMI_54h, and RPMI_168h compared to the LB groups. Additionally, we observed that Pal exhibited significantly higher levels in RPMI_54h and RPMI_168h, similar to Lpp, which showed a significant increase in RPMI_54h. In contrast to our findings, the results from previous studies showed a loss of function due to mutations or deletions of these gram-negative bacterial proteins (Deatherage et al., 2009; Schwechheimer et al., 2013; Schwechheimer and Kuehn, 2015). This could suggest that other proteins apart from Lpp and Tol-Pal complex proteins (Pal, TolA, and TolB) may participate in *E. coli* bMV biogenesis. However, future studies may also need to focus on whether TolB is essential for *E. coli* bMV biogenesis under LB and RPMI 1640 culture conditions. We also checked if *E. coli* experienced more stress when cultured in RPMI 1640 compared to LB groups. Interestingly, we found that four members of the universal stress protein Usp family (UspA, UspC, UspE, and UspF) (Nachin et al., 2005) were significantly higher expressed, and showed a more than 2-fold change in LB_54h and LB_168h but no difference in 8 h groups. However, the relationship between Usp and *E. coli* bMV biogenesis and secretion remains poorly understood.

### 4.4 Potential co-purified flagellum-like structure and consistent functional assay

We also discovered flagellum-like structures (Figure 2). Flagella, which are tiny hair-like structures, have the ability to detach from the cell surface either through mechanical force or active removal by the bacterium itself (Bahar et al., 2016). Flagella have been reported as a potential contamination in bMV isolation and density gradient centrifugation can potentially separate outer membrane vesicles from flagella contamination (Nevot et al., 2006; Klimentova and Stulik, 2015). However, flagella co-purification is still a controversial debate. Using 1.5 M ammonium sulfate precipitation with centrifugation as the method for isolating gram-negative bMVs, Blackburn et al. were the first to discover the reciprocal packaging of FimA (Type 1 Fimbriae Major Subunit) and FliC (Major Flagella Filament Structural Protein) within bMVs derived from wild-type *E. coli* K12 strain (Blackburn et al., 2021). As expected, co-purification of flagella and bMVs were consistently observed in their electron microscopy images using ultracentrifugation (Wai et al., 2003; Manabe et al., 2013; Kothary et al., 2017), which aligns with our own findings (Figure 2). Surprisingly, using sucrose gradient or OptiPrep^™^ (Iodixanol) to purify bMVs still revealed flagella (Bahar et al., 2016; Bitto et al., 2017). Furthermore, a previous report has demonstrated that flagella stimulation triggers the secretion of cytokines, including IL-1β and TNF-α, in monocytes (Ciacci-Woolwine et al., 1999). However, this study relied solely on centrifugation to purify flagella and ignored to mention the presence of bMVs. Our functional assay demonstrated comparable cytokine stimulation in THP-1 cells when treated with an equal number of bMVs (ratio 100 bMVs:1 cell) derived from different culture conditions (Figure 4). The co-purification of flagella could potentially explain why we were unable to observe any differences in the effects of bMVs from various culture conditions. On the other hand, we also noticed comparable rates of IL-1β and IL-8 secretion induction when treating with the six groups of *E. coli* bMVs alone, in comparison to treatment with 100 ng/mL LPS. However, little is known about whether flagella, bMVs, or LPS are the key for immune response, especially in the activation of monocytes/macrophages.

## 5 Conclusion

Our study provides an overview of the particle numbers, particle morphology and protein composition of *E. coli* bMVs isolated from two growth media, LB and RPMI 1640, at different points of the growth phase by conventional ultracentrifugation. Our study also confirmed that RPMI 1640 is an alternative particle free medium for *E. coli* cultivation and bMV collection. *Escherichia coli* bMVs isolated from RPMI 1640 showed a similar morphology and functionality compared to those from LB medium. From our proteome results, it was evident that the proteins associated with *E. coli* bMVs from the RPMI 1640 culture condition showed a stronger correlation with outer membrane pathways compared to those isolated from LB. The high yield of ribosomal proteins in *E. coli* bMVs may be linked to their role in promoting bacterial growth.

## Data availability statement

The mass spectrometric raw files and the MaxQuant output files have been deposited to the ProteomeXchange Consortium via the PRIDE partner repository, and can be accessed using the identifier PXD041301.

## Ethics statement

Ethical approval was not required for the studies on humans in accordance with the local legislation and institutional requirements because only commercially available established cell lines were used.

## Author contributions

MY: Conceptualization, Data curation, Formal analysis, Investigation, Methodology, Visualization, Writing – original draft. DC: Conceptualization, Data curation, Formal analysis, Investigation, Methodology, Visualization, Writing – original draft. MR: Funding acquisition, Project administration, Resources, Writing – review & editing. AM: Funding acquisition, Project administration, Writing – review & editing. FB: Project administration, Writing – review & editing. GS: Funding acquisition, Project administration, Writing – review & editing. CL: Data curation, Software, Writing – review & editing. CM: Data curation, Software, Writing – review & editing. BK: Writing – review & editing. CZ: Methodology, Writing – review & editing. LM: Funding acquisition, Project administration, Resources, Writing – review & editing. MP: Conceptualization, Funding acquisition, Project administration, Resources, Supervision, Writing – review & editing.

## Glossary

bMVs: bacterial membrane vesicles
CMO: CellMask^™^ orange
Cryo-EM: cryogenic electron microscopy
*E. coli*: *Escherichia coli*
EM: electron microscopy
EVs: extracellular vesicles
GOCC: gene ontology cellular component
*H. pylori*: *Helicobacter pylori*
IL: interleukin
*K. pneumoniae*: *Klebsiella pneumoniae*
LB: lysogeny broth/Luria–Bertani medium
LC–MS/MS: liquid chromatography–tandem mass spectrometry
LFQ: label-free quantification
MS: mass spectrometry
NTA: nanoparticle tracking analysis
OD_600_: optical density at 600 nm wavelength
PBS: phosphate buffered saline
PSD: particle size distribution
SD: standard deviation
TEM: transmission electron microscopy
TSA: tryptone soy agar
