# Supplementary figures and images for: The proteome of bacterial membrane vesicles in Escherichia coli—a time course comparison study in two different media
Source: Front Microbiol. 2024 Mar 6;15:1361270. doi: 10.3389/fmicb.2024.1361270 (PMC10954253; doi:10.3389/fmicb.2024.1361270)

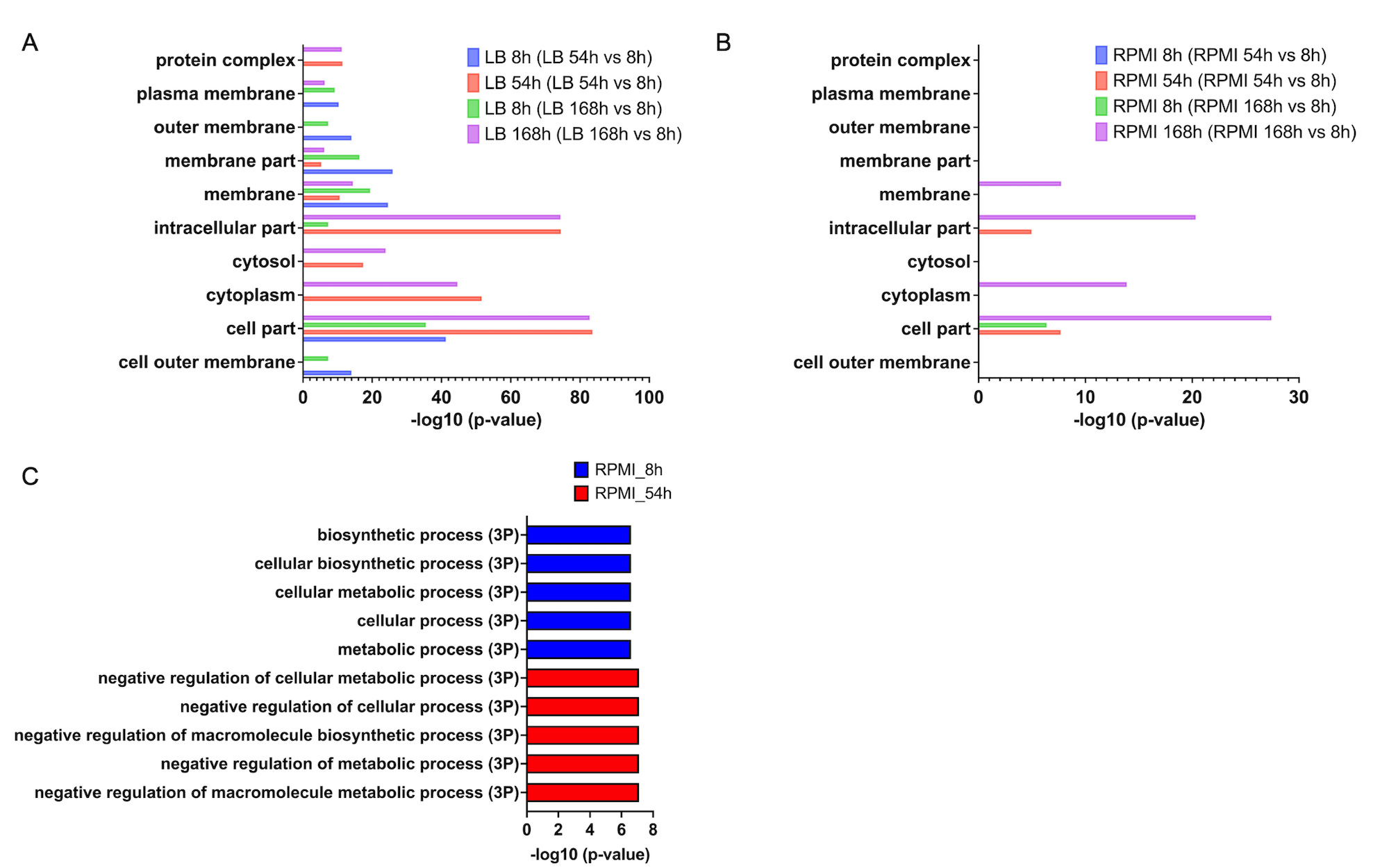

Supplement: Supplementary file 1 [file Image_1.tiff]
